# Serum Glucose-Dependent Insulinotropic Polypeptide (GIP) and Glucagon-Like Peptide-1 (GLP-1) in association with the Risk of Gestational Diabetes: A Prospective Case-Control Study

**DOI:** 10.1155/2020/9072492

**Published:** 2020-01-29

**Authors:** Maryam Mosavat, Siti Zawiah Omar, Sajad Jamalpour, Peng Chiong Tan

**Affiliations:** ^1^Department of Obstetrics & Gynaecology, Faculty of Medicine, University of Malaya, 50603 Kuala Lumpur, Malaysia; ^2^Department of Pharmacology, Faculty of Medicine, University of Malaya, Kuala Lumpur 50603, Malaysia

## Abstract

**Background:**

Defects in incretin have been shown to be related to the pathogenesis of type 2 diabetes. Whether such a deficiency happens in gestational diabetes mellitus (GDM) remains to be confirmed. We assessed the association of fasting glucose-dependent insulinotropic polypeptide (GIP) and glucagon-like peptide-1 (GLP-1) with GDM. We also studied the longitudinal circulation of these peptides during pregnancy and afterwards.

**Methods:**

53 women with GDM (30 managed with diet only (GDM-diet) and 23 treated with insulin (GDM-insulin)) and 43 pregnant women with normal glucose tolerance (NGDM) were studied, with GIP and GLP-1 levels measured at 24–28 weeks (E1), prior (E2) and after (E3) delivery, and postpuerperium (E4).

**Results:**

Basal GIP was shown to be low in GDM groups compared to NGDM in E1, and in E4 for GDM-diet. GLP-1 was low in GDM groups during pregnancy and afterwards. At E1, serum GIP and GLP-1 were inversely associated with GDM and participants with lower levels of GIP (<0.23 ng/mL) and GLP-1 (<0.38 ng/mL) had a 6 (95% CI 2.5-14.5)- and 7.6 (95% CI 3.0-19.1)-fold higher risk of developing GDM compared with the higher level, respectively. In the postpuerperium, when there is a drop in *β*-cell function, participants with previous GDM (pGDM) presented lower GLP-1 (in both GDM subgroups) and lower GIP in GDM-diet subgroup compared to controls.

**Conclusion:**

There is an independent, inverse association between fasting incretins and higher risk of GDM. Furthermore, lowered levels of these peptides may play an important role in the abnormality of glucose regulation following pregnancy.

## 1. Introduction

The gastrointestinal tract is the largest endocrine organ in the body, generating hormones that have significant signalling and sensing important roles in regulating body weight and energy expenditure [1]. Glucose-dependent insulinotropic polypeptide (GIP) and glucagon-like peptide-1 (GLP-1) are known incretin peptides secreted from the intestine in response to nutrient ingestion that stimulate insulin secretion together with hyperglycaemia [2, 3]. The physiological importance of islet-derived GLP-1 and GIP in insulin secretion has been previously studied [4], where their contribution to the regulation of *β*-cell mass is debated in the pathophysiology of type 1 and type 2 diabetes (T1&T2DM) [5, 6]. Impaired incretin effect has been reported in patients with T2DM, where it has been concluded that this deficit is the outcome of the diabetic state and not a primary pathogenic trait leading to T2DM [7]. Nevertheless, a study by Amato et al. [8] has suggested that fasting incretins play an important role in the pathophysiology of T2DM.

Pregnancy is a condition associated with the physiological and reversible expansion of *β*-cell mass in both animals and humans [9]. A study on the role of incretin peptides in islet adaptation to gestation, using incretin receptor knockout mice has revealed the significant role of GLP-1 in pregnancy-induced elevation of *β*-cell mass, mediated largely by local GLP-1 production in *α*-cells. However, that study also found that islet or K-cell-derived GIP is not essential for pregnancy-associated expansion of *β*-cell mass [9]. Gestational diabetes mellitus (GDM) is the most common medical complication during pregnancy and is defined as diabetes diagnosed during the gestational period that is not clearly overt diabetes [10, 11]. Higher fasting GLP-1 level in patients with GDM compared to pregnant women with NGDM have been observed by Cypryk et al. [12], where a lower but not significantly GLP-1 level has been reported by Lencioni et al. [13].

There is however paucity in information with regard to fasting gut peptide levels in pregnancy. We hypothesised that GDM pregnancies compared to NGDM will demonstrate impaired fasting levels of these peptides throughout pregnancy as the lack of these peptides can result in gestational hyperglycaemia. Further, beyond the pregnancy, low fasting gut peptide levels can provide early pathophysiologic insight into the transformation of GDM to T2DM later in life despite apparent normalisation of glucose tolerance in GDM after puerperium.

## 2. Material and Methods

This study was carried out in the Women and Children's Health Complex, University Malaya Medical Center (UMMC). The protocol of the present study was approved by the University of Malaya Medical Centre (UMMC) Ethics Committee (Ethics Committee Reference Number: 1052.8). A cohort of 434 patients was initially recruited at the time of GDM diagnosis (24-28 weeks of gestation). However, only 96 patients including 53 subjects diagnosed with GDM and 43 normal glucose tolerance pregnant control (NGDM) were entered in the longitudinal study as we were unable to obtain a fasting blood sample of all initially recruited participants at scheduled examination points. Furthermore, those that developed any pregnancy complications such as late-diagnosed GDM, preeclampsia, high blood pressure, eclampsia, and preterm labour were omitted from the longitudinal assessment [14]. The fasting maternal samples were collected at four points: (E1) 24-28 weeks of pregnancy at the time of OGTT, (E2) prior to parturition, (E3) early postpartum (24 hours after parturition), and (E4) 2-6 months of postpuerperium [14]. GDM was diagnosed as fasting plasma glucose (FPG) (≥5.1) and 75 g OGTT plasma glucose (≥7.8) [15].

Fasting glucose (FG) levels were measured using the glucose oxidase method (ADVIA® 2400 Clinical Chemistry System, Siemens, USA). A fasting level of serum total GIP, active GLP-1 (amide form), C-peptide, and insulin was determined using magnetic bead-based multiplex immunoassay, human diabetes panel (Bio-Plex Pro™, 171A7001M, USA) according to the manufacturer's protocol. The lower limit of quantitation (LLOQ) for GIP and GLP-1 was 11.2 and 31.3 pg/mL (0.0112 and 0.0313 ng/mL), and upper limit of quantitation (ULOQ) for GIP and GLP-1 was 22,895 and 16,000 pg/mL (22.895 and 16 ng/mL), respectively. The intra-assay coefficient of variation of GIP, GLP-1, insulin, and C-peptide was 2.47, 4.04, 3.04, and 5.9, respectively, where the interassay coefficient of variation was 3.08, 5.46, 2.55, and 2.33, respectively.

Body mass index (BMI) (weight (kg)/height (m^2^)) was measured at 24-28 weeks of gestation, prior to parturition, and postpuerperium. Homeostasis model assessment of *β*-cell function (HOMA-*β*) was calculated as (FI × 20) ÷ (FG‐3.5). Insulin resistance index (HOMA-IR) was calculated by the formula HOMA‐IR = [FI × FG]/22.5 [16]. HOMA model was derived from fasting blood glucose (FG) and fasting insulin (FI).

### 2.1. Statistical Analysis

The results were reported as mean ± standard error (SE), where a Kolmogorov–Smirnov test was used to assess the normality of data. Differences between groups were analysed using the Student *t*-test or Mann–Whitney *U* test. One-way ANOVA or the Kruskal–Wallis test was used for between-group comparisons in cases of more than two independent groups. Repeated measures ANOVA or Friedman's test was applied for within-group comparisons. In cases of sphericity assumption violation in repeated measures ANOVA, the Greenhouse–Geisser adjustment was used. Bonferroni post hoc analysis was used for pairwise comparisons within groups. Bivariate Spearman or Pearson was applied to assess correlations. The mean was considered as a cut-off point value, and logistic regression models were performed to compute crude/adjusted odds ratios (OR/aOR) and 95% confidence intervals (95% CI) comparing the risk of GDM among the two halves for serum GIP and GLP-1 concentrations. Skewed variables were log-transformed for skewed data. Statistical analysis was performed using IBM SPSS 20.0.

## 3. Results

Fifty-three subjects diagnosed with GDM, and forty-three normal glucose tolerance pregnant women (NGDM) were included from the cohort in this study. At the time of enrolment (E1), no significant differences were observed in the mean of maternal (*p* = 0.24) and gestational age (*p* = 0.72), prepregnancy (*p* = 0.40), pregnancy BMI (*p* = 0.88), systolic (SBP) and diastolic (DBP) blood pressure, and family history of diabetes between the studied groups. Normal pregnancy presented higher HDL and LDL levels (*p* = 0.02) compared to GDM (Table 1). As was expected, GDM pregnancy presented a higher fasting glucose level (5.0 vs. 4.24, *p* = 0.003), 2-hour OGTT (10.8 vs. 5.9, *p* = 0.005), insulin resistance index (HOMA-IR) (2.9 vs. 2.1, *p* = 0.03), and lower HOMA-*β* (8.46 vs. 13.53, *p* = 0.001) compared to normal glucose tolerant subjects. There was no difference between insulin (*p* = 0.72) and C-peptide (*p* = 0.39) in both groups.

The results of between- and within-group comparisons of GDM and control groups (NGDM) are presented in Table 2. In the longitudinal assessment, pregnancy diagnosed with GDM presented a higher level of FG compared to NGDM. There were significant changes in FG levels in both groups; however, its level increased immediately after delivery and remained unchanged in postpuerperium. Pregnant women of both groups had statistically similar fasting insulin and C-peptide levels during all points of examination. In both groups, serum insulin and C-peptide levels rose during pregnancy, reached a peak in the late pregnancy, and then decreased immediately after delivery. GDM groups presented lower HOMA-*β* compared to controls in E1 and E4.

Over the gestational period and postpuerperium, subjects diagnosed with GDM presented lower levels of GIP and GLP-1 compared to the control group, with the exception of E2 for GIP. In both pregnancy groups, concurrent with pregnancy development, GIP levels rose to hit their peak in late pregnancy, where levels decreased significantly after delivery and this reduction continued till postpuerperium. In contrast, GLP-1 levels decreased over the gestational period, and after delivery, this reduction continued in GDM subjects and then increased in postpuerperium.

Subsequently, in the third trimester (E2), the GDM group was divided into the GDM-diet subgroup (*n* = 30) and GDM-insulin subgroup (*n* = 23) (Table 3). Insulin treatment was initiated in GDM, where self-monitoring blood glucose indicated poor glycaemic control despite oral metformin therapy or diet. Pregnant women of the GDM-insulin subgroup had significantly higher FG compared to GDM-diet and NGDM in all examination points. In both GDM subgroups, FG levels decreased gradually over the pregnancy period, increased slightly in immediate postpartum, and then gradually decreased in postpuerperium. The level of insulin and C-peptide of all subgroups increased during pregnancy and gradually decreased after delivery. GDM-insulin subjects in E1 and both GDM subgroups in E4 presented lower HOMA-*β* compared to the normal group.

In the first examination, both GDM subgroups presented lower GIP and GLP-1 compared to NGDM. During the pregnancy, GIP levels increased in all subgroups, decreased gradually after delivery, and remained higher compared to the first examination. In postpuerperium, GIP level of the pGDM-diet group remained significantly low compared to the controls. In contrast with a significant decrease in the GLP-1 level of controls, GLP-1 level of each GDM subgroup remained unchanged during the gestational period. In immediate postpartum, GLP-1 level of pGDM subgroups decreased and then increased to the higher level; however, its levels remained statistically low compared to controls.

Regarding baseline variables (E1), serum GIP and GLP-1 were inversely associated with GDM. Participants with lower levels of GIP (<0.23 ng/mL) and GLP-1 (<0.38 ng/mL) had a 6- and 7.6-fold higher risk of developing GDM compared with the higher level, respectively. However, this relationship was relatively unchanged in GIP and became stronger in GLP-1 after adjustment for confounders including maternal age, gestational age, and BMI (aOR 5.7, 95% CI 2.3-14.3). Lower levels of *β*-cell function index (HOMA-*β*) were also inversely associated with the risk of GDM (0.88 (95% CI 0.81-0.96)) (Table 4). GLP-1 was directly correlated with GIP (*r* = 0.68, *p* < 0.001).

## 4. Discussion

In this study, we evaluated alterations in the basal level of serum glucose-dependent insulinotropic polypeptide (GIP) and glucagon-like peptide-1 (GLP-1) levels in women with and without GDM, in an attempt to investigate the role of these peptides in glucose homeostasis during pregnancy and afterwards. In general, low basal circulating level of incretins is due to rapid degradation by dipeptidyl peptidase-4 (DPP-4) [17]; however, lower amounts of these peptides are essential for glucoregulation and their consequent inhibitory effect on pancreatic *α*-cells [17].

This study showed that basal GIP and GLP-1 concentration plays a significant role in glucose homeostasis. GLP-1 is primarily synthesized by L-cells in the gastrointestinal tract, where it is influenced by ingested glucose and fatty acids or stimulated vagus nerve stimulation [18]. GLP-1 stimulates insulin secretion by *β*-cells in the pancreatic islets and inhibits glucagon secretion by *α*-cells [19]. GIP is mainly secreted by K-cells (in the mucosa of the duodenum and jejunum and the proximal portion of the ileum) and stimulates food intake-mediated insulin secretion by pancreatic *β*-cells [20]. It has been shown that GIP increases insulin's effect by directly changing target tissue sensitivity to insulin [21]. Furthermore, it has been suggested that GIP plays a role not only as an incretin hormone but also as a regulator of inflammation and insulin resistance [22]. GIP and GLP-1 share mutual characteristics as incretins, but they also possess distinct biological features [23]. Incretin peptides inhibit *β*-cell apoptosis and stimulate proliferation, resulting in the development of *β*-cell mass [17]. It has been shown that the transcription factor 7-like 2 (TCF7L2) gene is strongly associated with T2DM and GDM. Decreased TCF7L2 protein levels in T2DM correlated with the downregulation of GIP and GLP-1 receptors (GIP-R and GLP-1R), and impaired *β*-cell function [24, 25], lead to fasting and postprandial hyperglycaemia [26–28]. In the present study, we have shown that impaired fasting glucose at the time of GDM screening (24-28 weeks) is characterised by a reduced pancreatic *β*-cell function evaluated by HOMA-*β* and decreased insulin sensitivity evaluated by HOMA-IR. Interestingly, we found that low fasting levels of GIP and GLP-1 were inversely associated with higher risks of GDM in pregnancy. Pregnant subjects with a low level of GIP and GLP-1 were about 6- and 7.6-fold, respectively, at higher risk of GDM. As shown previously, GDM results from reduced pancreatic *β*-cell function [29]. Similarly, the importance of fasting incretin peptides has also been studied in patients with T2DM, and it was proposed that the reduced fasting incretin levels in the presence of worsening fasting glucose is secondary to weakened fasting *β*-cell function associated with increased *α*-cell activity [8]. The authors of that study suggested that early assessment of basal incretin level would be useful in the diagnosis of T2DM. A deficit in the regulation of these peptides has also been proposed, involved in the glucose homeostasis in T2DM, where it has been suggested as a novel possibility for treating subjects with T2DM [30].

In the longitudinal assessment of the present study, with progression in pregnancy, GLP-1 concentrations remained statistically unchanged in GDM groups, whereas its level decreased significantly in controls. The level of circulating GLP-1 did not differ between the GDM-insulin subgroup and nondiabetic pregnant women, whereas GDM-diet presented a lower level of this peptide compared to controls. This result indicates the effectiveness of exogenous insulin administration in the regulation of GLP-1 in GDM. Normal pregnancy is associated with insulin resistance and with pregnancy progression, where insulin sensitivity may gradually decline to 50% of the normal expected value [31, 32]. It has been proven that enlarged *β*-cell mass is an adaptation to progressive insulin resistance that develops during gestational period [33]. In line with our findings, lower fasting GLP-1 level have been reported in association with GDM and T2DM [34, 35]. In contrast, no significant difference in basal GLP-1 level of GDM patients and controls has been reported previously [13, 36]; this discrepancy between results may be due to the small sample size that was used in these studies. Furthermore, GLP-1 concentrations of GDM subgroups showed a reduction immediately after delivery and then increased in the late postpuerperium. However, after delivery, its level increased gradually and reached to the highest level in the late postpuerperium compared to women with GDM in their pregnancy. From this result, we postulate that low concentrations of GLP-1, disregarding the mode of treatment with diet or insulin during pregnancy, may indicate early abnormality of glucose regulation and progression to T2DM.

Furthermore, with progression in pregnancy, we observed a gradual progress in *β*-cell function (HOMA-*β*) occurring with insulin resistance progress in all subgroups. Furthermore, GIP levels increased with pregnancy advancement and reached a peak in late pregnancy. Our study postulates that elevations in basal GIP levels during pregnancy in GDM subgroups may play an effective role in controlling fasting glycaemia and insulin resistance. In the late postpuerperium, GIP continued to stay in the low level in women with previous GDM treated with diet but not in insulin-treated subgroup. Furthermore, we found that insulin treatment during GDM could control GIP levels in the postpuerperium period and reduce its contribution in the pathogenesis of T2DM. However, further research is warranted to investigate the effect of insulin treatment on fasting GIP level.

In summary, this study has presented longitudinal circulation levels of fasting GIP and GLP-1 during pregnancy, after parturition and postpuerperium. From the current study, we postulate that lower levels of these peptides play a major role in the increased risk of GDM and dysregulation of glucose after pregnancy.

## Figures and Tables

**Table 1:** Baseline characteristics of participants (mean ± SE).

Participants' age (year)	
GDM	33.2 (0.6)
NGDM	32.1 (0.8)
Gestational age (week)	
GDM	25.8 (0.2)
NGD	25.9 (0.2)
Prepregnancy BMI (kg/m^2^)	
GDM	26.1 (0.8)
NGDM	25.2 (0.7)
Pregnancy BMI (kg/m^2^)	
GDM	29.3 (0.7)
NGDM	29.1 (0.9)
HOMA-IR	
GDM	2.9 (0.3)
NGDM	2.1 (0.2)^∗^
SBP (mmHg)	
GDM	110.70 (1.14)
NGDM	109.40 (1.46)
DBP (mmHg)	
GDM	66.23 (1.19)
NGDM	2.26 (0.07)
HDL (mmol/L)	
GDM	2.05 (0.06)
NGDM	2.26 (0.07)^∗^
LDL (mmol/L)	
GDM	2.59 (0.14)
NGDM	3.02 (0.11)^∗^
Cholesterol (mmol/L)	
GDM	5.81 (0.14)
NGDM	6.11 (0.14)
TG (mmol/L)	
GDM	2.68 (0.36)
NGDM	2.14 (0.13)

^∗^ *p* value < 0.05 difference between groups.

**Table 2:** Between- and within-group comparisons of subjects with gestational diabetes mellitus (GDM, *n* = 53) vs. control group (NGDM, *n* = 43) (mean ± SE).

	E1	E2	E3	E4
FG (mmol/L)				
GDM	5.00 ± 0.22	4.66 ± 0.15	5.01 ± 0.18^b^	4.66 ± 0.08
NGDM	4.24 ± 0.06	4.36 ± 0.13	4.60 ± 0.12^a^	4.35 ± 0.08
*p* value	0.003	0.15	0.08	0.009
Insulin (ng/mL)				
GDM	0.45 ± 0.03	0.71 ± 0.09^a^	0.50 ± 0.08^b^	0.41 ± 0.03
NGDM	0.47 ± 0.06	0.61 ± 0.07^a^	0.40 ± 0.04^b^	0.45 ± 0.03
*p* value	0.72	0.40	0.34	0.28
HOMA-*β*				
GDM	8.46 ± 0.67	10.36 ± 1.45	6.23 ± 0.86	7.56 ± 0.62
NGDM	13.53 ± 1.45	14.92 ± 2.03	8.12 ± 1.28^a^	13.71 ± 1.75^c^
*p* value	0.001	0.17	0.18	0.001
C-peptide (ng/mL)				
GDM	1.13 ± 0.09	1.86 ± 0.16^a^	1.62 ± 0.21^b^	1.14 ± 0.07
NGDM	1.01 ± 0.10	1.44 ± 0.16^a^	1.25 ± 0.14	1.59 ± 0.28
*p* value	0.39	0.07	0.16	0.09
GIP (ng/mL)				
GDM	0.19 ± 0.01	0.54 ± 0.06^a^	0.36 ± 0.04^a,b^	0.29 ± 0.03^a,b^
NGDM	0.28 ± 0.01	0.53 ± 0.09^a^	0.53 ± 0.11^a^	0.38 ± 0.03^a^
*p* value	<0.001	0.57	0.007	<0.001
GLP-1 (ng/mL)				
GDM	0.34 ± 0.01	0.33 ± 0.01^a^	0.30 ± 0.01^a,b^	0.38 ± 0.02^b,c^
NGDM	0.47 ± 0.02	0.37 ± 0.01^a^	0.48 ± 0.01^b^	0.50 ± 0.01^b^
*p* value	<0.001	0.003	<0.001	<0.001

^∗^ *p* value < 0.05 difference between groups. ^a^*p* < 0.05 compared to examination 1. ^b^*p* < 0.05 compared to examination 2. ^c^*p* < 0.05 compared to examination 3.

**Table 3:** Between- and within-group comparisons of GDM-diet (*n* = 23) and GDM-insulin (*n* = 30) subgroup vs. control group (NGDM) (*n* = 43).

	Group	E1	E2	E3	E4
FBG (mmol/L)	GDM-diet	4.52 ± 0.11	4.37 ± 0.13	4.73 ± 0.21	4.62 ± 0.11
	GDM-insulin	5.67 ± 0.47^∗^^,†^	5.05 ± 0.30^∗^^,†^	5.41 ± 0.30^∗^	4.70 ± 0.10^c^
	NGDM	4.24 ± 0.06	4.36 ± 0.13	4.60 ± 0.12	4.35 ± 0.08

Insulin (ng/mL)	GDM-diet	0.45 ± 0.05	0.67 ± 0.14	0.50 ± 0.11	0.40 ± 0.03
	GDM-insulin	0.44 ± 0.04	0.77 ± 0.11^a^	0.52 ± 0.14	0.41 ± 0.05^b^
	NGDM	0.47 ± 0.06	0.61 ± 0.07	0.40 ± 0.04	0.45 ± 0.03

HOMA-*β*	GDM-diet	9.79 ± 0.83	10.75 ± 1.12	6.91 ± 1.38	7.77 ± 0.93^∗^
	GDM-insulin	6.65 ± 1.01^∗^	9.91 ± 2.27	5.27 ± 0.71	7.29 ± 0.80^∗^
	NGDM	13.53 ± 1.45	14.92 ± 2.03	8.12 ± 1.28	13.71 ± 1.75

C-peptide (ng/mL)	GDM-diet	1.17 ± 0.13	1.82 ± 0.20^a^	1.61 ± 0.23	1.12 ± 0.10^b^
	GDM-insulin	1.07 ± 0.10	1.92 ± 0.25	1.64 ± 0.40	1.17 ± 0.11^b^
	NGDM	1.01 ± 0.10	1.44 ± 0.16	1.25 ± 0.14	1.59 ± 0.28

GIP (ng/mL)	GDM-diet	0.20 ± 0.01^∗^	0.54 ± 0.09^a^	0.34 ± 0.05^a^	0.24 ± 0.03^b,^^∗^
	GDM-insulin	0.18 ± 0.02^∗^	0.54 ± 0.08^a^	0.38 ± 0.08^a^	0.34 ± 0.0 4^a^
	NGDM	0.28 ± 0.01	0.53 ± 0.09	0.53 ± 0.11	0.38 ± 0.03

GLP-1 (ng/mL)	GDM-diet	0.36 ± 0.001^∗^	0.34 ± 0.01	0.30 ± 0.01^a,^^∗^	0.39 ± 0.03^∗^
	GDM-insulin	0.33 ± 0.011^∗^	0.31 ± 0.01	0.29 ± 0.01^a,b,^^∗^	0.36 ± 0.03^c,^^∗^
	NGDM	0.47 ± 0.02	0.37 ± 0.01	0.48 ± 0.01	0.50 ± 0.01

Pairwise analysis comparison (Bonferroni post hoc adjustment) following one-way ANOVA or Kruskal–Wallis test. ^a^*p* value < 0.05 compared to examination 1. ^b^*p* value < 0.05 compared to examination 2. ^c^*p* value < 0.05 compared to examination 3. ^†^*p* value < 0.05 compared to GDM-diet. ^∗^*p* value < 0.05 compared to control.

**Table 4:** Binary logistic regression analysis for the prediction of gestational diabetes mellitus (*n* = 96).

		GDM	
		No (*n* = 43)	Yes (*n* = 53)	Crude OR (95% CI)	Adjusted OR (95% CI)
		*N* (%)	*N* (%)		
GLP-1 (ng/mL)					
Half 1	<0.38	10 (21.3)	37 (78.7)	7.6 (3.0-19.1)	11.5 (3.8-34.8)
Half 2	≥0.38	33 (67.3)	16 (32.7)	Referent	Referent
GIP (ng/mL)					
Half 1	<0.23	12 (24.5)	37 (75.5)	6.0 (2.5-14.5)	5.7 (2.3-14.3)
Half 2	≥0.23	31 (66.0)	16 (34.0)	Referent	Referent
*p* value				<0.001	0.001
HOMA-*β*					
Half 1	<9.04			2.64 (1.1-6.2)	2.48 (1.0-6.00)
Half 2	≥9.04			Referent	Referent
*p* value				0.027	0.044

OR (95% CI) adjusted for maternal age, gestational age, and BMI. ^∗^*p* value < 0.05.

## Data Availability

The data used to support the findings of this study are available from the corresponding author upon request.
